# A Case of* Acinetobacter* Septic Pulmonary Embolism in an Infant

**DOI:** 10.1155/2016/5241571

**Published:** 2016-07-26

**Authors:** Poonam Wade, Anitha Ananthan, Jane David, Radha Ghildiyal

**Affiliations:** Department of Pediatrics, Topiwala National Medical College and BYL Nair Charitable Hospital, Mumbai 400008, India

## Abstract

*Case Characteristics*. An 11-month-old girl presented with fever and breathlessness for 5 days. Patient had respiratory distress with bilateral coarse crepitations. Chest radiograph revealed diffuse infiltrations in the right lung with thick walled cavities in mid and lower zone. Computed tomography showed multiple cystic spaces and emboli. Blood culture grew* Acinetobacter* species.* Intervention*. Patient was treated with Meropenem and Vancomycin.* Outcome*. Complete clinical and radiological recovery was seen in child.* Message*. Blood cultures and CT of the chest are invaluable in the evaluation of a patient with suspected septic pulmonary embolism. With early diagnosis and appropriate antimicrobial therapy, complete recovery can be expected in patients with septic pulmonary embolism.

## 1. Introduction

Though* Acinetobacter* is not an infrequent culprit for nosocomial infection in children, its occurrence as pulmonary embolism is a rare event [1]. Septic pulmonary embolism (SPE) is an uncommon condition that typically presents with fever, respiratory symptoms, and lung infiltrates [2]. The common radiographic findings are presence of bilateral multiple cavitary nodules in the lung [3]. In children septic emboli may be caused by osteomyelitis, cellulitis, urinary tract infection, jugular vein or umbilical thrombophlebitis, right sided bacterial endocarditis, or septic thrombophlebitis [4]. We hereby present a case of septic pulmonary embolism caused by* Acinetobacter* species in an infant.

## 2. Case Report

An 11-month-old boy presented with high grade fever for 15 days and breathlessness for 4 days. On examination, this febrile child (axillary temperature: 38°C) had tachycardia (pulse rate: 124/min), tachypnea (respiratory rate: 64/min), and respiratory distress in the form of subcostal and intercostal retractions. He was pale and had bilateral coarse crepitations in infra-axillary and infrascapular region.

**Figure 1:**
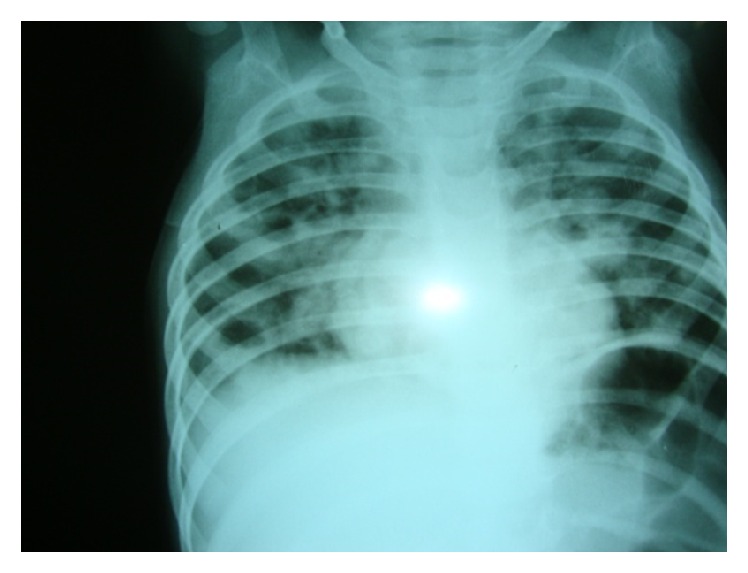
Chest radiograph revealed diffuse infiltration in right lung with thick walled cavities in mid and lower zone. Infiltrations were also seen in left upper parahilar region.

**Figure 2:**
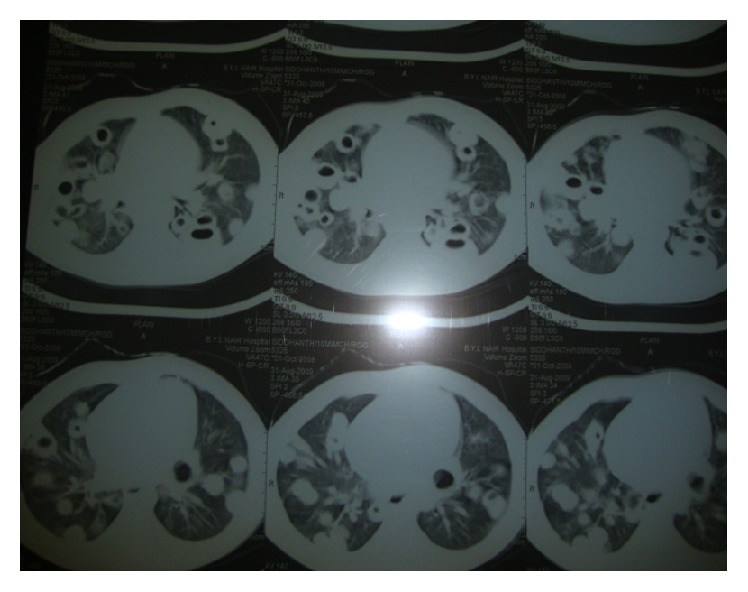
Computed tomography showing bilateral cavitary nodules and emboli.

Laboratory investigations revealed anemia (Hb: 7.5 g/dL), leukocytosis (WBC count: 30,600/cu. mm, with 56% polymorphs), and thrombocytosis (platelet count: 600000/cu. mm). The chest radiograph revealed diffuse infiltration in right lung with thick walled cavities in mid and lower zone. Infiltration was also present in the left upper parahilar region (Figure 1). Making a diagnosis of bronchopneumonia, therapy was initiated with intravenous antibiotic therapy with Ceftriaxone 100 mg/kg/day and Amikacin 15 mg/kg/day. Vancomycin 60 mg/kg/day was added on day 2 suspecting staphylococcal pneumonia as the causative organism due to the presence of cystic spaces seen on chest radiograph.* Acinetobacter* species was isolated from blood culture sample collected on admission. The organism was sensitive to Meropenem. In view of lack of any clinical improvement, therapy with Meropenem 40 mg/kg/day was initiated. Computed tomography (CT) of the chest was performed to rule out possibility of lung abscesses, which showed bilateral multiple cavitary nodules and emboli (Figure 2). Patient's fever and respiratory distress decreased after 3 weeks of antibiotics. Antibiotics were continued for 28 days.

## 3. Discussion

This communication describes a child with community-acquired pneumonia with septic embolization due to* Acinetobacter* species. Despite extensive literature search we did not come across such a case described from India.

SPE is an uncommon and serious disorder associated with significant morbidity and mortality. Clinical and radiological features at presentation are nonspecific and the diagnosis of this disorder is frequently delayed [2]. SPE has been associated with risk factors such as IV drug use, pelvic thrombophlebitis, and suppurative processes in the head and neck and presence of an indwelling catheter and immunocompromised patients [2]. In SPE, the embolic blood clot along with microorganism causes an infarction in the pulmonary vasculature which can lead to focal abscess.

Our patient presented with fever as well as signs and symptoms of pneumonia. As chest radiograph showed infiltration and thick walled cavities, patient underwent a CT scan, which showed bilateral multiple round, cavitary nodules in peripheral portions of both lungs. SPE is characterized by scattered, well-defined peripheral nodules, in various stages of cavitation, with feeding vessels, and subpleural wedge-shaped densities, with or without septic infarcts [5]. It is important to diagnose SPE because it is associated with not only increased mortality and increased hospital stay but also complications such as abscesses, empyema, and bronchopleural fistula which require different therapeutic interventions.

The cause of SPE in our patient was* Acinetobacter* infection though staphylococcus is the most commonly isolated organism [6].* Acinetobacter*, an aerobic gram-negative coccobacillus, is an important pathogen in nosocomial pneumonia. But rarely it can also cause community-acquired pneumonia. In* Acinetobacter* genus, the* Acinetobacter baumannii* species is a common cause of community-acquired pneumonia in tropical/subtropical region and other places with warm and humid season [7]. In the present study, we described a patient with community-acquired pneumonia with septic pulmonary embolism caused by* Acinetobacter* species. Strains of* Acinetobacter baumannii* causing community-acquired infections are usually susceptible to Aminoglycosides, extended-spectrum Penicillin, Ceftazidime, Quinolone, Imipenem, and Ciprofloxacin [7]. Our patient responded to Meropenem and Vancomycin with radiological resolution.

## 4. Conclusion

*Acinetobacter* species is an uncommon but important cause of severe community-acquired pneumonia. It should be suspected in patients who present with fulminant course. Early diagnosis of septic pulmonary embolism is important to prevent complications like abscesses, empyema, and bronchopleural fistula. A combination of third generation Cephalosporin and Aminoglycoside may be an appropriate empirical therapy. With early diagnosis and appropriate antimicrobial therapy, complete resolution can be seen in patients with septic pulmonary embolism and in children.
